# Sedimentary basins investigation using teleseismic P‐wave time delays

**DOI:** 10.1111/1365-2478.12747

**Published:** 2019-03-18

**Authors:** Nicola Piana Agostinetti, Francesca Martini

**Affiliations:** ^1^ Department of Geodynamics and Sedimentology University of Vienna Vienna 1090 Austria; ^2^ Geophysics Section, School of Cosmic Physics Dublin Institute for Advanced Studies Dublin Ireland; ^3^ Tullow Oil plc Applied Geophysics Team Dublin Ireland

**Keywords:** Passive method, Signal processing, Wave

## Abstract

Passive seismic methods have been proven successful in recent years at retrieving information about the large‐scale structure of a sedimentary basin. These methods are based on ambient noise recordings, and local and distant (teleseismic) earthquake data. In particular, it has been previously observed that the arrival time of teleseismic P‐waves recorded inside a sedimentary basin shows time delays and polarization that both strongly depend on the basin properties and structure. In this paper, we present a new methodology for determining seismic P‐wave velocity in a sedimentary basin, based on the time delay of a teleseismic P‐wave travelling through the low‐velocity basin infill, with respect to a teleseismic wave recorded outside the basin. The new methodology is developed in a Bayesian framework and, thus, it includes estimates of the uncertainties of the P‐wave velocities. For this study, we exploit synchronous recordings of teleseismic P‐wave arrivals at a dense linear array of broadband seismic stations, using data from two teleseismic events coming from two different incoming angles. The results obtained by the new proposed methodology are successfully compared to classical cross‐correlation measurements, and are used to infer properties of a sedimentary basin, such as the basin bounding fault's geometry and the average P‐wave velocity of the sedimentary basin fill.

## 1 INTRODUCTION

Defining the structure and the elastic properties of a sedimentary basin is fundamental in hydrocarbon exploration. Active seismic is the most widely used method for obtaining a picture of the subsurface, and for positioning and drilling of wells. In recent years, passive seismic methods have been used as complementary techniques in frontier exploration. These methods are based on the passive recording of natural occurring vibrations, either earthquakes or ambient noise (Biryol *et al*. 2013; Behm, Leahy and Snieder 2014). Ambient noise measurements can be used to constrain the dispersion of the surface waves passing through the sedimentary strata and therefore, by inversion methods, to map the structure of a basin (Martini *et al*. 2013, 2015, and references therein). Moreover, seismic waves generated by large magnitude earthquakes occurring at very large distance from the target area (so‐called *teleseisms*, with sources located at more than 3000 km from the recording seismic stations) also carry valuable information for constraining the elastic properties of the basin of interest (Srinivas *et al*. 2013; Licciardi and Piana Agostinetti 2017; Liu, Persaud and Clayton 2018; Piana Agostinetti, Martini and Mongan 2018). In particular, the arrival time of teleseismic P‐waves recorded inside a sedimentary basin displays time delays and polarization that both strongly depend on the basin properties and topography (e.g. Schmandt and Clayton 2013; Hofstetter and Dorbath 2014; Bao and Niu 2017).

In this study, we present a new methodology for measuring seismic P‐wave velocity ($V_{P}$) in a sedimentary basin, based on time delays cumulated by teleseismic P‐waves travelling through soft sediments. In more detail, we use recordings of teleseismic P‐wave arrivals at a dense linear array of broadband seismic stations, deployed normal to the sedimentary basin axis. The new methodology is developed in a Bayesian framework and, thus, it includes estimates of the uncertainties on the P‐wave velocities. We illustrate the new methodology using field measurements from a sedimentary basin within the Kenya rift. Results are compared to classical cross‐correlation measurements and discussed in light of their integration in a wider set of tools that can be used in basin exploration.

## 2 DATA AND METHODS

**Figure 1:**
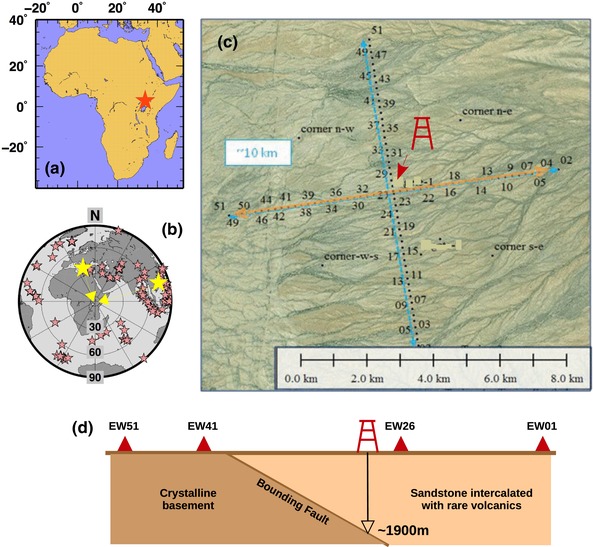
(a) Regional map with the position of the temporary seismic network (red star). (b) Distribution of the teleseismic events (Mw≥5, red stars) that occurred during the deployment of the temporary passive seismic network. The two yellow stars indicates the two teleseismic events analysed in this study. (c) Map of the study area in false colours, with the geometry of the passive seismic network. Black dots indicate the locations of a seismometer (numbers indicate station name). Blue lines defines the length of the two passive seismic profiles. An orange double‐head arrow indicates the seismic stations used in this study. The position of the well is also shown. (d) Sketch of the geometry of the basin along the East–West profile, with the depth of the basement drilled at station EW26.

**Figure 2:**
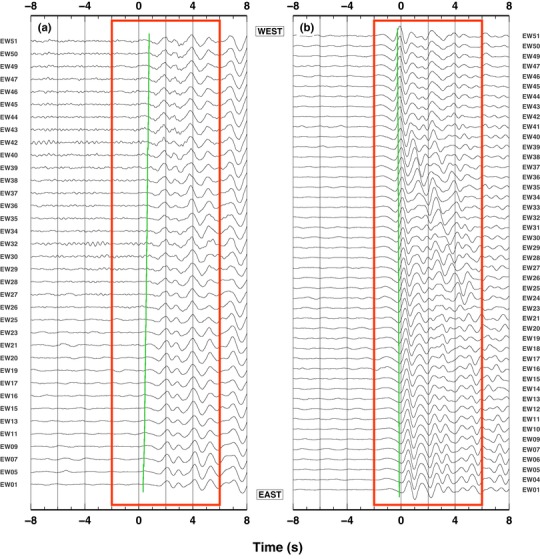
Examples of teleseismic waveforms: (a) updip arrivals; and (b) along‐strike arrivals. P‐wave arrival at all the stations that recorded such teleseism. Theoretical arrival‐time at the first station is set a *t* = 0, throughout all figures reporting waveforms data. A red box shows the 8‐s‐long time‐window used for computing cross‐correlation and likelihood value. A green line indicates theoretical P‐wave arrival time computed using IASP91 model (Kennett and Engdahl 1991). In this case, for the updip arrivals (panel ‘a'), the stations to the East (e.g. EW01) displays theoretical earlier arrivals (about 0.5 s) with respect to the stations on the West (e.g. EW51).

We use teleseismic P‐waves recorded by a temporary seismic deployment, across one of the young sedimentary basins within the East African Rift valley (Fig. 1). The dense (200 m spacing), linear array of 50 3‐component broadband seismic stations strikes normal to the basin axis, with some stations deployed on the footwall of the basin fault and the rest directly on top of the Neogene sediments. The stations are named EW01–EW51, with stations EW51 at the western end of the profile. Stations EW51–EW41 lie outside the basin, on basement; stations EW41 marks the position of the outcropping basin bounding fault, with the remaining stations sitting on top of the sedimentary basin. A well drilled to basement at ∼1900 m depth is located along the profile (close to station EW26). To illustrate our new methodology, we select two moderate ($M_{w}$ = 5.0−5.5) teleseismic events recorded from the majority of the seismic stations (Fig. 2, https://sim-crust.dias.ie/RF_as_an_exploration_tool.html). Such events have completely different source regions, so that back‐azimuthal directions of the incoming P‐waves are parallel and perpendicular to the linear array (Table 1). Such choice allows to test the stability of the technique for different source‐array geometry. For the analysis, we only use the data recorded by the vertical component, because we are focusing on the teleseismic P‐wave arrival, which is mainly recorded on such component. Moreover, the vertical component is more representative of the teleseismic source function, with less contamination of P‐to‐s converted phases generated by the receiver‐side structure, when compared to the horizontal ones (Mostafanejad and Langston 2017). Theoretical computations indicate that, for an event occurring in the parallel direction to the linear array, the time delay difference between the first and the last stations in the array could be as large as about 0.3–0.4 s (Fig. 2). Such theoretical time delays need to be considered for obtaining unbiased results and, thus, traces are aligned with their theoretical P‐wave arrival times (obtained using the IASP91 Earth model by Kennett and Engdahl 1991). Following a general approach to teleseismic waveform analysis, we remove high and low frequency using a bandpass filter between 0.5 and 5 Hz (e.g. Rawlinson and Kennett 2004).

**Table 1:** Information on the teleseismic events used in this study.

Event	OT Date	OT Time	Lat	Lon	*D* (km)	$M_{W}$	Baz	Dist	N. traces
1410030805	14/10/03	80547.01	11.35	122.32	30.	5.5	78.6	84.4	36
1410242343	14/10/24	234315.01	38.92	21.13	1.	5.3	340.2	41.9	46

Baz and dist indicate back‐azimuth and epicentral distance with respect to the seismic network, respectively. *D* is focal depth in kilometres. N. traces reports the number of available vertical traces for each event. Anthropic seismic noise reduced the numbers of available traces for the first event. Due to the different epicentral distance and backazimuth, the results obtained for the two events should be carefully combined in case of complex structures.

We develop a new methodology, based on Monte Carlo sampling, to compute the absolute time delays with respect to a given point of the linear array. The new methodology follows the workflow developed by VanDecar and Crosson (1990)(VDC90, hereinafter). Briefly, for each teleseism, the VDC90 method: (a) computes cross‐correlation time delays for each pair of stations in a network, (b) discards results from poorly correlated waveforms with *ad hoc* criteria for removing outliers and (c) computes absolute time‐delays for each station, imposing a Zero‐Average to the sum of the absolute time delays. This approach has been applied in several studies and gives stable results under different conditions of event magnitude, network geometry and tectonic settings, from local to global scale (e.g. Gibbons and Ringdal 2006; Schmandt and Humphreys 2010). However, two main pitfalls arise in the VDC90 method. First, outliers are discarded or repicked by adopting user‐defined criteria, to avoid “cycle‐skipping” issues (i.e. where self‐similar portions of the waveforms are misidentified by the cross‐correlation function, which is, by definition, not sensitive to waveform amplitude). Second, uncertainties of the absolute time delays are not directly estimated from the data uncertainties, but computed from assumed error models (usually a least square approximation).

We modified the VDC90 method by introducing a hierarchical Bayes approach to the analysis of each pair of teleseismic records, based on a Monte Carlo sampling of the model space (Malinverno and Briggs 2004; Bodin *et al*. 2012; Piana Agostinetti, Giacomuzzi and Malinverno 2015). Here, the model space is represented by the time delay between the two traces and by an additional hyper‐parameter that scales the covariance matrix of the residuals. Hierarchical Bayes allows to estimate uncertainties in model parameters (here, in the estimated time delays) directly from the data (Malinverno and Briggs 2004). Moreover, methodologies based on Monte Carlo sampling are less prone to be trapped in local maxima (e.g. avoid “cycle‐skipping” issues) and can easily map global maxima of the likelihood surface (Sambridge 1998). The computational cost of Monte Carlo algorithms is their main drawback. In this case, computations have been performed on a 4‐CPUs laptop in less than 1 hour. In case of a large number of teleseisms, time delays for each single teleseism can be easily computed on a computer cluster, and thus, computation time can be split across several CPUs.

**Figure 3:**
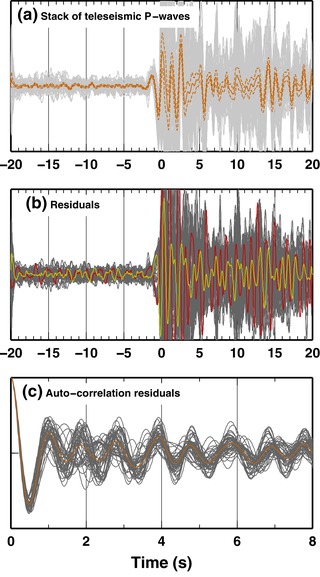
Example of data analysis for one teleseismic event. (a) Stack of all traces, aligned to their theoretical P‐wave arrival times. An orange solid line shows the stack value; dotted line is the standard deviation of the stack. Grey lines are single records of the teleseismic P‐wave at each station. (b) Residuals for each single record from the stack value (grey lines). Red and a yellow lines display the residuals for the first and the last station along the linear profile, respectively. (c) Autocorrelation of the residuals. Each grey line shows the autocorrelation of the residuals for one station. An orange line indicates the average of all autocorrelation curves, used for computing realistic covariance matrix of the errors.

A fundamental point in Monte Carlo methods is the definition of the likelihood function, which is based on assumed error statistics. Here we assume the errors in the waveforms to be Gaussian distributed with known, non‐zero, correlation (i.e. a full covariance matrix). Using un‐correlated error statistics (i.e. a diagonal Covariance matrix) clearly violates the postulate of the waveforms being band‐limited signals (even more in our case, where we apply a further bandpass filter) and has been proven to lead to unrealistic results (e.g. Figure S6 in Chai *et al*. 2017). Thus, we estimate the full covariance matrix from the autocorrelation of the residuals, following the approach described in Piana Agostinetti and Malinverno (2018). Firstly, we compute the stack of the aligned waveforms, together with its standard deviation (Fig. 3a). For each trace, residuals from the stack are then extracted (Fig. 3b). As expected, the residuals calculated for the two distant stations display opposite trends (red and yellow lines in Fig. 3b). Finally, we compute an average autocorrelation function of the residuals (Fig. 3c). The average autocorrelation function and the standard deviation of the stacks are used to compose the full covariance matrix of the residuals (see equation (1) in Piana Agostinetti and Malinverno 2018). Our approach allows an estimate of realistic covariance matrix, which is used to compute the Likelihood function for a Gaussian distributed variable (equation (9) in Malinverno 2002).

## 3 RESULTS

**Figure 4:**
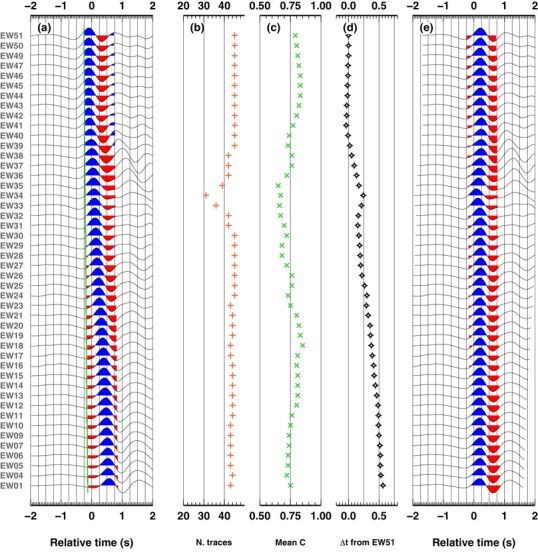
Results adopting VDC90's method, for the first teleseism. (a) Zoom of the original first P‐wave arrival for each seismic station. Colours are used to highlight the first second after theoretical P‐wave arrivals. The green line indicates the theoretical P‐wave arrival times. (b) Number of cross‐correlation values selected after removing outliers, for each station. (c) Average cross‐correlation value for each station. (d) Absolute time delay for each station, relative to station EW51. (e) Aligned first P‐wave arrival for each seismic station, using the value in panel (d) and theoretical P‐wave arrival estimates.

We first show the full results obtained for one teleseism, using both the VDC90 method and our new approach. We compute the absolute time delays adopting the VDC90 method, to have coherent results for a direct comparison with the results obtained with our method. In Fig. 4, we show the results obtained using the VDC90 method. Pre‐processing and post‐processing traces are shown in panels (a) and (e) and visually confirm the validity of the results. In panel (b), we show the number of cross‐correlation values obtained for each station, after removing outliers. As expected, where waveforms are less similar to the stack (e.g. trace EW34), a larger number of outliers is found (which yields to the discarding of about 15 traces when computing single station time delay from the reference station, i.e. here station EW51). This observation is confirmed by the low average values of the cross‐correlation function for the same stations (Fig. 4c). Figure 4(d) reports the absolute time delays for each station, relative to the last station of the linear array (EW51, which lies outside the sedimentary basin). Results clearly show an increasing trend in the time delays towards the centre of the basin with decreasing station number, due to the P‐waves travelling through the sediments. Due to their location on basement rock, the stations EW50–EW41, deployed outside the basin, do not display any relevant time delays.

**Figure 5:**
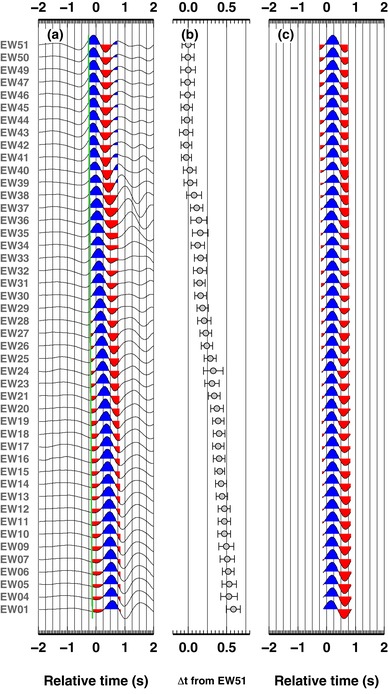
Results using Bayesian inferences, for the first teleseism. (a) As in Fig. 5(a). (b) Absolute time delay relative to station EW51, computed using the Bayesian approach, which includes estimates of time delay uncertainties. (c) As in Fig. 5(e), traces are aligned using the value in panel (b) and theoretical P‐wave arrival estimates.

In Fig. 5, we report the results obtained, for the same event, with our new approach. Pre‐processing and post‐processing traces are shown in panels (a) and (c) and again confirm the validity of the results and their similarity to the results obtained with the VDC90 methodology. Absolute time delays relative to station EW51 (Fig. 5b) are also similar to the VDC90 results (Fig. 4d), and confirm the clear trend of increasing time delays from stations EW51 towards EW01 (Eastward). In this case, each absolute time‐delay has an associated error estimate. In our new approach, we follow the assumption in the VDC90 method, that is the two stages workflow and the imposed constraint of zero‐average time delay; therefore, our error estimates should not be considered absolute measurements, but realistic measures of the relative errors between different stations. In fact, with the adoption of a full covariance matrix for the errors in the residuals, such errors are derived directly from the data.

**Figure 6:**
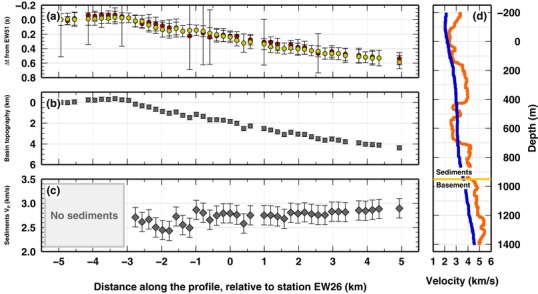
(a) Bayesian results for the two events (red, updip arrival, and yellow, along‐strike arrival, symbols) (b) Estimates of Basin shape beneath each single station based on the results in panel (a). Basin thickness is computed by adopting a fixed velocity model. Vp value for the basement is assumed as 4.5 km/s. A Vp value for sedimentary rocks is estimated from the depth of the basement drilled at station EW26 and the time‐delay computed for the same station (see text). (c) $V_{P}$ velocity for sedimentary rocks, adopting a defined basin fault geometry. Here, fault emergence is fixed at −3.0 km along the profile, fault dip is 30° and the profile is considered normal to the strike of the fault (Piana Agostinetti *et al*. 2018). $V_{P}$ value for the basement is assumed as 4.5 km/s. (d) Sonic‐log (orange line) from a borehole located close to the seismic profile, indicating seismic velocity for the sediments and basement rocks. Stacking velocity used in active seismic migration is also shown (blue line). Reported sonic‐log does not belong to the well drilled close to station EW26.

The results obtained for the two teleseisms are used to infer the elastic properties of the sedimentary basin. Assuming vertical ray‐propagation, the time‐delay for a P‐wave arrival time between *i*th station inside the basin and one station outside can be computed as:

(1) $${\Delta t}_{i}=\frac{H_{i}}{V_{P,\text{sed}}}-\frac{H_{i}}{V_{P,\text{bas}}},$$

where $H_{i}$ represents the depth of the basin beneath *i*th station and $V_{P,\text{bas}}$ and $V_{P,\text{sed}}$ are P‐wave velocity in the basement and sediments, respectively. Teleseismic P‐wave time delays could, in principle, be used for a more general geophysical inversion to derive both the basin topography and the distribution of the elastic parameters within the basin. Here we make use of equation (1) and additional constraints from a three‐dimensional (3D) active seismic survey and a ∼1900‐m‐deep well, positioned along the profile (at station EW26), to make simplified inferences on the basin bounding fault geometry and the P‐wave velocity ($V_{P,\text{sed}}$) within the sedimentary basin fill. In Fig. 6(a), we present the absolute time delays obtained for the two teleseismic events. At each station, the obtained P‐wave time delays for both events are extremely similar, confirming that the back azimuthal direction of the incoming P‐wave does not influence the estimates in this geological/structural setting. This fact implies that a substantial number of events can be used for this analysis, even if data from a short deployment (less than 1 month) are available. More complex subsurface structures might be investigated analysing separately arrivals from different directions. In Fig. 6(b), the thickness of the sedimentary column is measured, beneath each station. Here we assume that (a) the P‐wave velocity in the basement is known, and fixed at $V_{P,\text{bas}}=4.5$ km/s, as for metamorphosed crystalline rocks in the area (Fig. 6d); (b) the sedimentary column is homogeneous; and (c) the depth of the basement is known at the well position, about 1900 m. Given the frequency observed in the teleseismic wave, the assumption of an homogeneous sedimentary layer is suitable (https://sim-crust.dias.ie/RF_as_an_exploration_tool.html). Using our absolute time delay value for station EW26 (dt = 0.24237 s), installed at the well location, the P‐wave velocity for the sedimentary basin infill is estimated to be 2.91 km/s (Fig. 1d). Such value is used at all other stations to attempt to map the basin structure. As seen in Fig. 6(d), the values we used for basement rocks (*Vp* = 4.5 km/s) and sediments (Vp = 2.9 km/s) fall within the values found in borehole measurements. Moreover, those values are close to the one used in the active seismic data processing (stacking values, also reported in Fig. 6d). Results clearly show the dipping interface between sediments and basement, tracing it to the Eastward end of the profile. The interface seems to be continuous to station EW01, where active seismic data cannot image it, likely due to the presence of volcanics within the sediments. Figure 6(c) show the complementary computation of the P‐wave velocity for sedimentary rocks. In this case, we assume that: (a) P‐wave velocity in the basement is known, at 4.5 km/s; (b) the sedimentary column is homogeneous and (c) the basin bounding fault geometry is also known, dipping 30° towards the end of the profile (from active seismic data Piana Agostinetti *et al*. 2018). From these constraints, the average P‐wave velocity for the sedimentary column is computed for all the stations within the basin. Results display values in‐between 2.4 and 2.9 km/s consistent with estimates for wet sandstones type of rock (Kassab and Weller 2015), as well as with the range of velocities observed in the area by the processing of 3D active seismic data and with sonic logs recorded at the local wells (Fig. 6d).

A more refined use of the absolute time delays data can be developed, for example, to jointly model both the fault geometry and the elastic properties, based on a larger number of teleseismic observations. However, our simple exercises demonstrate the potential of teleseismic P‐wave for delivering independent measurements of elasticity in a sedimentary basin. The absolute time delays defined here can be considered a point measurement of P‐wave velocity and can be used in parallel with other complementary approaches, that is modelling of surface waves dispersion in ambient noise measurement (Martini *et al*. 2013, 2015) and receiver function analysis (Licciardi and Piana Agostinetti 2017; Piana Agostinetti *et al*. 2018). In fact, such tools are mostly sensitive to S‐wave velocity variations in the rocks, as such an initial constraint on the P‐wave velocity would strongly benefit the inversion of the data. Thus, the observations presented in this study naturally fill the gap in passive seismic modelling of sedimentary basins, ensuring an independent local measurement of the P‐wave velocity.

## 4 CONCLUSIONS

In this paper, we present a new methodology for determining seismic P‐wave velocity in a sedimentary basin, based on time delays measured in teleseismic P‐waves travelling through a basin sedimentary infill. The new methodology is developed in a Bayesian framework and, thus, includes estimates of the uncertainties on the P‐wave velocities. For this study, we use recording of two teleseismic events with different back azimuths at a dense linear array of broadband seismic stations.

The results obtained by the new proposed methodology:

1. display comparable values to more classical approaches, like cross‐correlation measurements, without imposing user‐defined criteria for data selection. This fact allows an estimation of uncertainties in the measurements directly from the raw‐waveforms;
2. are used to infer topography of the sedimentary basin. Retrieved basin geometry includes the correct dip for the bounding fault, from its emergence at the surface to the end‐point of the seismic profile, where it is noteworthy that the active seismics do not clearly image it.
3. are exploited to infer independent measurements of P‐wave velocity in the sedimentary column beneath each seismic station. P‐wave velocity in the sandstone is constrained to 2.4 and 2.9 km/s, close to laboratory measurements for wet sandstone samples and in agreement with the average velocities in the area as per active seismic data and well data.

This work clearly demonstrates the potential of using teleseismic P‐waves to ensure an independent local measurement of the P‐wave velocity. Such measures can be used in parallel to other complementary techniques, such as modelling of surface waves dispersion (Behm *et al*. 2014) and receiver function analysis (Leahy, Saltzer and Schmedes 2012).
